# Genome-Wide Characterization of *DrRALF* Genes in Yam (*Dioscorea rotundata*) Reveals Their Potential Roles in Tuber Expansion and the Gibberellin Response

**DOI:** 10.3390/ijms26136151

**Published:** 2025-06-26

**Authors:** Qinghua Qiao, Furui Sheng, Wei Qiao, Shanshan Li, Liying Wang, Dong Xiao, Longfei He

**Affiliations:** 1 Key Laboratory of Mountain Biodiversity Conservation, Education Department of Guangxi Zhuang Autonomous Region, Yulin Normal University, Yulin 537000, China15064128510@163.com (F.S.); 19151568220@163.com (L.W.); 2 National Demonstration Center for Experimental Plant Science Education, College of Agriculture, Guangxi University, Nanning 530004, China; 3 College of Plant Sciences, Tibet Agricultural and Animal Husbandry University, Linzhi 860000, China; qiaovv77@163.com

**Keywords:** gibberellin (GA), gene expression, rapid alkalinization factor (RALF), tuber expansion, yam, yeast one-hybrid assay

## Abstract

Yam (*Dioscorea* spp.) provides various nutritional and medicinal benefits, including a high starch content, dietary fiber, essential micronutrients, and bioactive compounds. The molecular mechanisms underlying tuber expansion have not yet been clarified. Rapid alkalinization factor (*RALF*) genes, which mediate various processes in plants, are thought to contribute to the regulation of tuber growth; however, their role in yam development, especially in gibberellin (GA)-mediated processes, remains unclear. Here, we characterized seven *DrRALF* genes in the yam genome. Analysis of gene duplication demonstrated that the expansion of *DrRALF* genes was primarily driven by whole-genome duplication or segmental duplication. Phylogenetic analysis revealed that *DrRALF* genes were concentrated in specific clusters, indicating that their functions are relatively conserved. *DrRALF5* was specifically expressed in the roots, and *DrRALF2*, *DrRALF3*, *DrRALF4*, and *DrRALF6* were highly expressed in flowers. *DrRALF1*, *DrRALF2*, *DrRALF3*, *DrRALF4*, *DrRALF5*, and *DrRALF6* were shown to play a role in tuber expansion. Subsequent qRT-PCR validation of four selected *DrRALF* genes confirmed the regulation of *DrRALF2*, *DrRALF4*, *DrRALF5*, and *DrRALF6* by GA and PP333 (paclobutrazol, a GA biosynthesis inhibitor). Yeast one-hybrid assays further showed that the *DrRALF6* promoter region interacted with the GA-signaling protein, DrDELLA1. Our findings provide novel insights into the regulatory network controlling yam tuber expansion, especially through the interaction between *DrRALF6* and GA signaling pathways. Our results clarify the molecular mechanisms involved in tuber growth and propose a promising strategy for improving yam production through genetic manipulation of the GA-RALF signaling pathway.

## 1. Introduction

Yam (*Dioscorea* spp.) is a monocotyledonous tuber crop, and over 600 species have been described in the genus *Dioscorea*. Among tuber crops, yam ranks fourth in terms of global production volume, following *Solanum tuberosum* (potato), *Manihot esculenta* (cassava), and *Ipomoea batatas* (sweet potato), and it contributes approximately 10% to the total global production of roots and tubers [1]. In addition to its richness in starch, sugars, essential minerals, proteins, and vitamins, yam tubers also serve as a significant source of secondary metabolites, including steroidal saponins, diterpenoids, and alkaloids. Yam thus serves as a vital food source as well as an important medicinal crop [2,3]. The growth and expansion of yam tubers are key determinants of overall yield and also influence physical attributes such as tuber size and texture; clarifying the regulatory mechanisms underlying tuber expansion is thus essential for enhancing yam production and quality. Plant hormones, especially gibberellins (GAs), are critically important for regulating tuber expansion. GAs affect tuber growth and development through both direct and indirect mechanisms, including the regulation of cell elongation, division, and carbohydrate metabolism [4,5,6,7,8]. Previous studies indicate that GAs play a complex and stage-dependent role in tuber development. For example, GAs have been shown to negatively regulate the tuber expansion process, as the exogenous application of GA4/7 promotes stolon elongation and delays tuber formation [9]. In Jerusalem artichoke, GA3 levels are higher before tuber formation, which supports stolon growth, but decrease during tuber growth, and GA3 is negatively correlated with dry matter and sugar accumulation in tubers [10]. The exogenous application of GA increases the yield of yam tubers and promotes the formation of axillary bulbils [6,11]. The “GA-GID1-DELLA” signaling pathway, in which the DELLA protein serves as the central hub, is the currently recognized GA signaling pathway [12,13]. Tuber initiation in yam is regulated by the GA-GID1-DELLA module [6,14].

Rapid alkalinization factors (RALFs) are small, secreted cysteine-rich peptides found across various plant species. They induce rapid alkalinization in tobacco suspension cell cultures [15]. RALF members interact with various receptor protein kinases to regulate numerous biological processes, including plant growth, flowering, fruit maturation, and stress responses. Previous studies of *Arabidopsis* have demonstrated that AtRALF regulates plant growth by regulating cell proliferation and plays key roles in root, pollen tube, and fruit development [16,17,18,19,20,21]. RALFs have also been shown to be involved in pollen maturation in broccoli and *Primula vulgaris* [22,23]. In rubber trees, HbRALF3 and HbRALF19 regulate latex metabolism by affecting the pH of rubber latex [24]. In *Solanum chacoense*, fruit development and maturation are closely associated with RALFs, and *ScRALF3* silencing decreases seed production [25,26]. In legumes, seven GmRALFs are likely responsible for the release of rhizobia from cortical cells, indicating that they mediate nodule formation [27]. Although the roles of RALF peptides in regulating key developmental processes across diverse plant species are well-established, RALF peptides in yam (*Dioscorea* spp.) have not yet been functionally characterized. Given that yam is a nutritionally and medicinally important tuber crop, elucidating the regulatory functions of RALF peptides in tuber expansion is crucial for enhancing our knowledge of the molecular mechanisms of tuber development. Moreover, investigating the potential interplay between RALF-mediated pathways and gibberellin signaling may reveal novel regulatory nodes that govern tuber growth. These insights not only address a critical knowledge gap but also lay the groundwork for precision breeding strategies aimed at enhancing yam yield, quality, and stress resilience, which can ultimately contribute to sustainable agriculture and global food security.

Here, we identified *RALF* genes in yam by performing BLASTP (TBtools v1.108) searches against the yam genome and analyzed their potential functions in yam growth and development, including during tuber expansion. We also explored interactions between RALF family members and the GA signaling pathway. A visual summary of the experimental design and analytical pipeline is provided in Supplementary Figure S1. Our findings enhance our understanding of the regulatory mechanisms of *RALF* genes in yam tuber expansion, which may have implications for the development of strategies to enhance yam yield and quality and studies on hormonal regulation in other tuber crops. The primary aim of this study was to identify and characterize *RALF* genes in yam (*Dioscorea rotundata*) at the genome-wide level and explore their expression patterns during tuber development and in different plant tissues to provide preliminary insights into their potential roles in growth and development.

## 2. Results

### 2.1. Genomic Analysis of DrRALFs

*RALF* genes in yam were identified using a bidirectional BLAST strategy. *AtRALF* sequences were used as query sequences against the *Dioscorea* genome, which yielded seven candidate genes (Table 1). These candidate genes were subsequently validated through comparison with the SwissProt protein database, which confirmed that all seven candidates were *RALF* genes.

**Figure 1:**
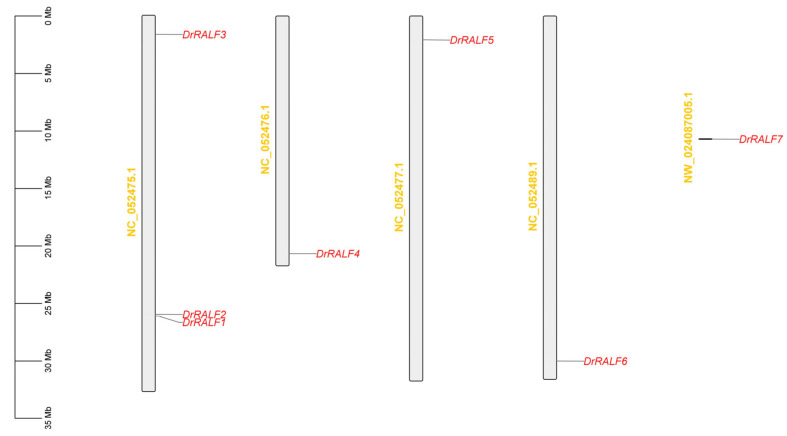
Chromosomal mapping of *DrRALFs* in the yam genome. Six *DrRALF* genes (*DrRALF1*–*DrRALF6*) were distributed across chromosomes with NC identifiers, and *DrRALF7* was located on an unanchored scaffold labeled with an NW identifier. Red text represents the individual gene names of *DrRALFs*, while yellow text indicates the corresponding chromosomes. The chromosomal positions were visualized using TBtools (v1.108). The scale on the left indicates chromosome length in megabases (Mb). Only chromosomes containing *DrRALF* gene loci are are shown.

**Figure 2:**
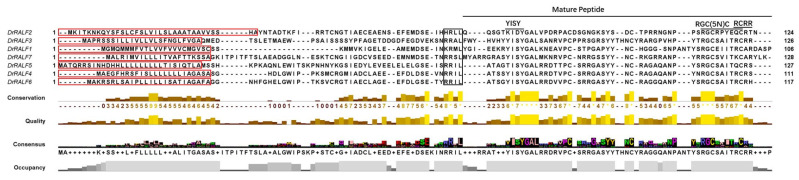
Amino acid sequence alignment of DrRALFs. The red box highlights the predicted signal peptide, and the black box marks the proteolytic cleavage site. Based on the characteristic features of plant RALFs, the mature domain of DrRALFs was identified, which includes the N-terminal YISY motif, the C-terminal RGC(5N)C motif, and the RCRR motif. Consensus represents the most frequent amino acid at each position across all aligned sequences, providing a visual summary of conserved residues. Occupancy indicates the frequency of each amino acid at every position, reflecting the variability or conservation of that position within the aligned sequences. Quality denotes the confidence in the alignment accuracy at each position, with higher quality indicating more reliable and precise alignments. Conservation illustrates the degree of evolutionary conservation, with higher conservation indicated by brighter shading and lower conservation by darker shading.

The seven identified genes were mapped to five chromosomes in the yam genome (Table 1 and Figure 1); based on their chromosomal locations, they were named *DrRALF1* to *DrRALF7*. Protein parameters of the seven DrRALFs were predicted by Protein Parameter Calc. The *DrRALF* protein precursors comprised 106–128 amino acids, with molecular weights between 11.63 and 14.31 kDa. The theoretical isoelectric points (pI) of the proteins ranged from 6.55 to 9.83, which corresponds to the pH at which the protein carries no net charge [28]. Most DrRALFs were found to be hydrophilic, with GRAVY values ranging from −0.596 (DrRALF2) to 0.029 (DrRALF1); negative values were observed for most proteins, suggesting they were predominantly hydrophilic (Table 2). The seven *DrRALF* protein precursors all contained signal peptides; five of them contained a conserved Arg-Arg (RR) motif, which was used as the cleavage site for site-1 protease (S1P). The mature *DrRALF* peptides consistently featured a conserved RGC(5N)C motif at the C-terminus. Additionally, five DrRALFs displayed a conserved YISY motif at the N-terminus, except for DrRALF2 and DrRALF5. The RCRR motif was highly diverse among DrRALF proteins (Figure 2).

We analyzed the duplication events of *DrRALFs* to investigate their evolutionary relationships. The results indicate that *DrRALF4/5/6* arose from whole-genome duplication (WGD) or segmental duplication, *DrRALF3/7* underwent dispersed duplication events, and *DrRALF1/2* are singletons (Supplementary Table S4).

**Figure 3:**
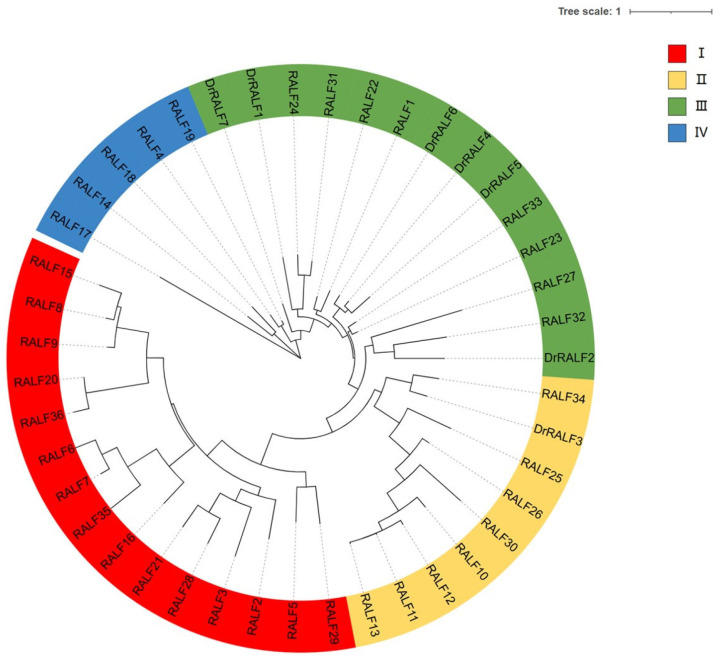
Phylogenetic tree of *RALFs* in yam and *Arabidopsis*. The proteins are classified into four major clades (I–IV), indicated by red, yellow, green, and blue. *DrRALF* genes represent yam sequences, while *RALF* genes without a prefix correspond to *Arabidopsis thaliana*.

A phylogenetic analysis was performed on *DrRALFs* as well as *Arabidopsis RALFs*. The *RALF* sequences were classified into four distinct clades, which were denoted as I, II, III, and IV, comprising 15, 9, 14, and 5 members, respectively. The *DrRALFs* were predominantly clustered in clade III, comprising six members, and clade II contained only a single *DrRALF*. Notably, no *DrRALFs* were identified in clades I and IV. Furthermore, a paralogous gene cluster (*DrRALF4/5/6*) was identified, which has been previously characterized to arise from WGD or segmental duplication events. In addition, the phylogenetic proximity between *DrRALF3* and *RALF34*, as well as that between *DrRALF2* and *RALF32*, indicates that these pairs are likely orthologous genes (Figure 3).

### 2.2. Cis-Acting Element Analyses of Yam DrRALFs

**Figure 4:**
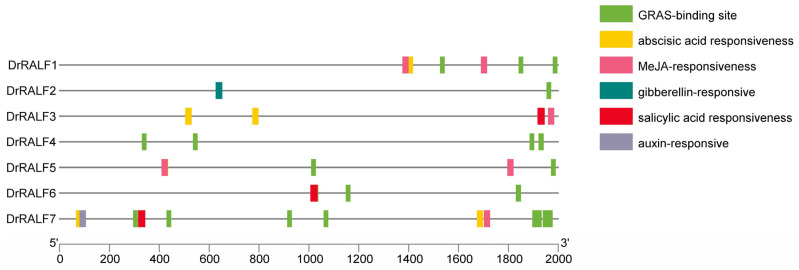
*Cis*-regulatory elements in the *DrRALF* promoter regions in yam.

The 2000 bp upstream promoter sequences of *DrRALF*s were retrieved from the yam genome, and their *cis*-regulatory elements were analyzed using the PlantCARE database, which allows their regulatory potential to be determined (Supplementary Table S1 and Figure 4). *Cis*-regulatory elements associated with plant hormone responsiveness were extensively distributed throughout the promoter regions of *DrRALFs* (Figure 4). The promoter regions of the seven *DrRALF* genes contained a total of 17 *cis*-regulatory elements linked to plant hormone responsiveness. The *DrRALF7* promoter contained the most *cis*-acting elements, which included two abscisic acid (ABA)-responsive elements, one auxin-responsive element, one methyl jasmonate (MeJA)-responsive element, and one salicylic acid (SA)-responsive element. This was followed by the *DrRALF3* promoter, which contained *cis*-regulatory elements responsive to ABA, MeJA, and SA. Both *DrRALF1* and *DrRALF5* contained two hormone-responsive elements, specifically those linked to ABA and MeJA. By contrast, *DrRALF2* and *DrRALF6* each contained only a single hormone-responsive element.

We analyzed the GRAS-binding sites within the promoter regions of *DrRALFs*. The results indicated that six of the seven *DrRALF* genes contained a total of 23 GRAS-binding sites in their promoter regions. Among these, the promoter of *DrRALF7* contained the most GRAS-binding sites (10), followed by *DrRALF4* (4 sites), *DrRALF1* and *DrRALF6* (each with 3 sites), *DrRALF5* (2 sites), and *DrRALF2* (1 site). No GRAS-binding sites were detected in the *DrRALF3* promoter.

### 2.3. Expression Patterns of DrRALFs in Different Organisms

**Figure 5:**
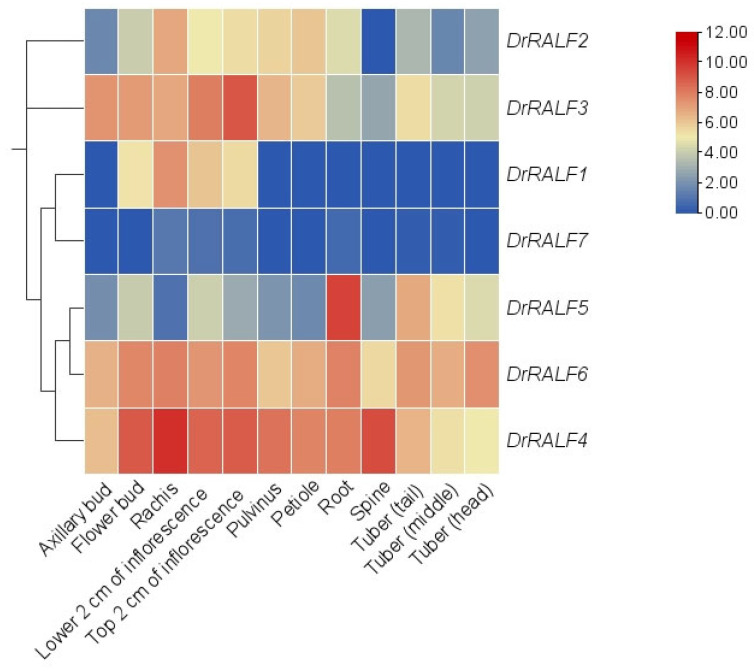
Heatmap of *DrRALF* expression levels across 12 different tissues in yam.

The expression of *DrRALFs* was examined using RNA-seq data, and we focused on their tissue-specific expression patterns and roles during tuber development. *DrRALF7* exhibited very low expression levels across all 12 tissues and was undetectable in some tissues. *DrRALF1* was exclusively expressed in flowers. By contrast, *DrRALF2*, *DrRALF3*, *DrRALF4*, *DrRALF5*, and *DrRALF6* were highly expressed across the 12 tissues, with the exception of *DrRALF2* in spines and *DrRALF5* in the rachis. The expression levels of *DrRALF2*, *DrRALF3*, *DrRALF4*, and *DrRALF6* were the highest in flowers, whereas that of *DrRALF5* was the highest in the roots (Figure 5 and Supplementary Table S5).

**Figure 6:**
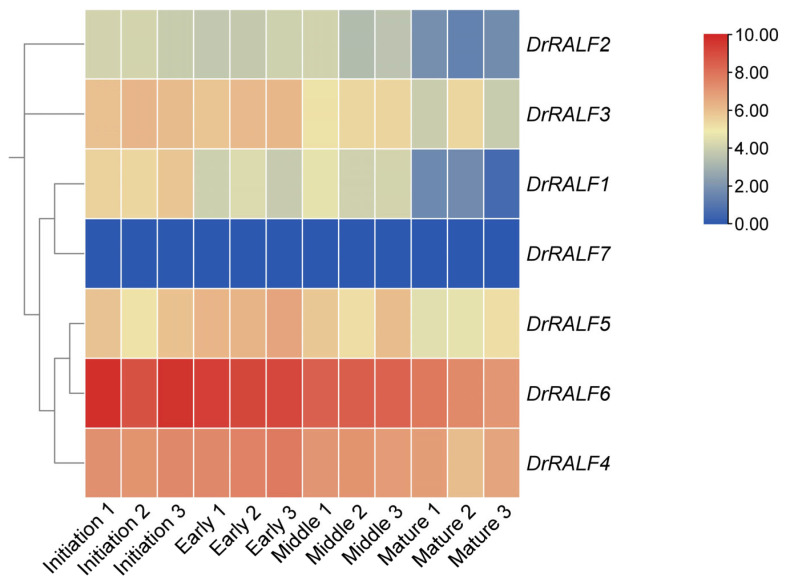
Heatmap of *DrRALF* expression levels at different developmental stages of yam tubers.

The expression profiles of *DrRALFs* were further investigated across all stages of tuber development. *DrRALF7* expression was undetectable across all stages of tuber development. In contrast, the transcript levels of *DrRALF1*, *DrRALF2*, *DrRALF3*, and *DrRALF6* became progressively down-regulated during tuber development. The expression of *DrRALF4* and *DrRALF5* peaked during early tuber development, and this was followed by a decline in transcript abundance as the tubers matured (Figure 6 and Supplementary Table S6).

### 2.4. qRT-PCR Revealed the Role of DrRALFs in Tuber Expansion

**Figure 7:**
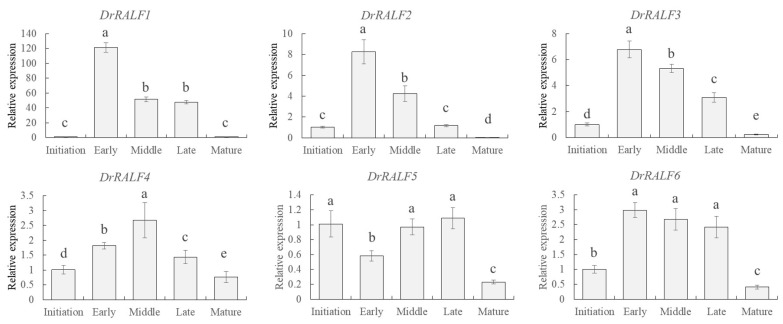
The expression levels of *DrRALFs* during tuber expansion. Different letters (a, b, c, d, e) denote significant differences (*p* < 0.05).

To further elucidate variation in the expression of *DrRALF* genes during tuber development, qRT-PCR was used to analyze their expression in yam tubers at five distinct developmental stages: the tuber initiation stage, early expansion stage, mid-expansion stage, late expansion stage, and maturation stage. As *DrRALF7* expression was not detected, our analysis focused exclusively on *DrRALF1*–*6*. Consistent with the RNA-seq data, the expression of *DrRALF1/2/3/6* progressively declined from the early expansion stage to the maturation stage. However, in contrast to the RNA-seq results, the expression of these genes was lower during the tuber initiation stage than during the early expansion stage. The expression profile of *DrRALF4* was distinct, and its expression gradually increased from the initiation stage to the middle stage; it then declined steadily thereafter. In contrast, the expression of *DrRALF5* was the highest during the late expansion stage (Figure 7).

**Figure 8:**
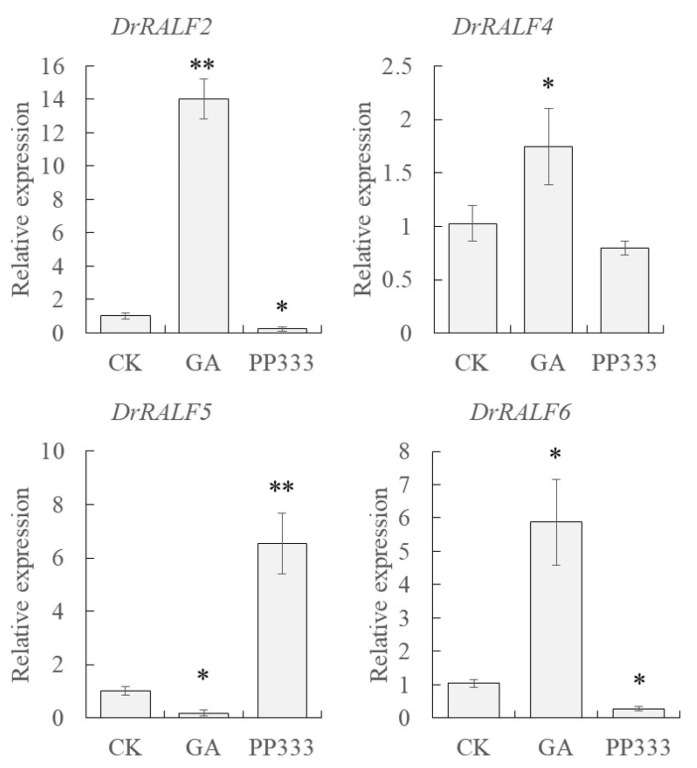
Expression patterns of *DrRALF2/4/5/6* genes in yam tubers in response to exogenous GA and PP333 treatments. Asterisks (*) denote significant differences (*p* < 0.05), while double asterisks (**) denote highly significant differences (*p* < 0.01).

Through an integrative analysis combining bioinformatics and qRT-PCR, four *DrRALF* genes (*DrRALF2/4/5/6*) were found to exhibit significant stage-specific expression patterns during yam tuber development. The presence of GRAS-binding sites in the *cis*-regulatory elements of the promoters reflects the potential functions of these genes in GRAS-mediated regulatory pathways. According to these findings, these four genes were designated as key candidates and subjected to subsequent functional and mechanistic studies. We subsequently analyzed the effects of GA and PP333 treatments on their expression patterns. Exogenous GA treatment led to the significant up-regulation of *DrRALF2/4/6* expression in yam tubers, and the expression of *DrRALF5* was significantly down-regulated. In contrast, treatment with exogenous PP333 reduced the expression levels of *DrRALF2/6*, and *DrRALF5* expression was significantly enhanced (Figure 8).

### 2.5. Characterization of the Secretory Properties of DrRALFs

**Figure 9:**
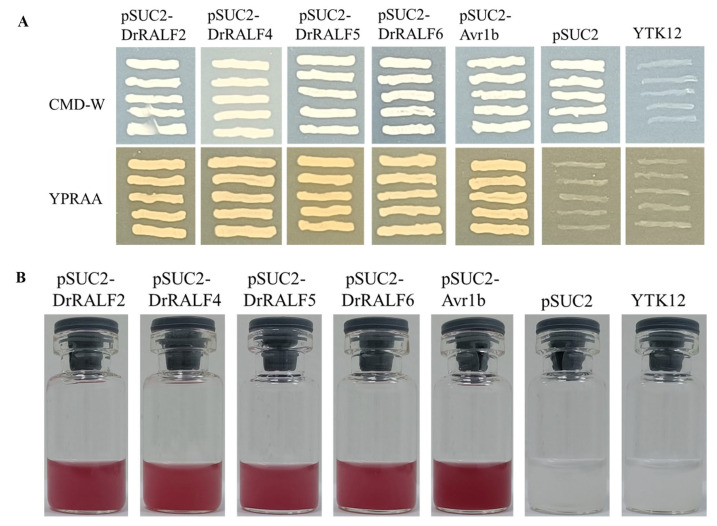
Validation of the secretory properties of DrRALF2/4/5/6. (**A**): Assessment of the secretory function of DrRALF2/4/5/6 using the yeast signal peptide tracking system. (**B**): Confirmation of the secretory activity of DrRALF2/4/5/6 through the TTC color reaction.

The pSUC2 yeast secretion system was used to validate the secretory functions of DrRALF2/4/5/6. Recombinant plasmids containing pSUC2-DrRALF2, pSUC2-DrRALF4, pSUC2-DrRALF5, and pSUC2-DrRALF6 were generated and transformed into YTK12; pSUC2 and pSUC2-Avr1b were used as the negative and positive control, respectively. The results demonstrated that the negative control failed to grow on YPRAA plates, whereas yeast cells expressing pSUC2-DrRALF2, pSUC2-DrRALF4, pSUC2-DrRALF5, and pSUC2-DrRALF6 could grow normally on both CMD-W and YPRAA plates (Figure 9A). The secretory characteristics of DrRALFs were further validated using the TTC color reaction. The results showed that yeast strain YTK12, which contains recombinant plasmids encoding the signal peptides of pSUC2-DrRALF1, pSUC2-DrRALF2, pSUC2-DrRALF5, pSUC2-DrRALF6, or the positive control pSUC2-Avr1b, secreted functional invertase, which is capable of hydrolyzing sucrose into monosaccharides. These monosaccharides reacted with TTC to generate water-insoluble red triphenylformazan, which provides strong biochemical evidence confirming the secretory function of DrRALF2/4/5/6 (Figure 9B).

### 2.6. Validation of the Interaction Between DrRALFs and DrDELLA1 Through Yeast One-Hybrid Assay

The promoter regions of *DrRALF2/4/5/6* were cloned into the pHIS2 vector to determine whether DrDELLA1 regulates *DrRALF2/4/5/6* expression (Supplementary Files S1–S4 and Figures S2–S5), and the *DrDELLA1* coding sequence was inserted into the pGADT7 vector (Supplementary File S5 and Figure S6). The background screening assay revealed that the transformants harboring pHIS2-DrRALF2+pGADT7, pHIS2-DrRALF4+pGADT7, pHIS2-DrRALF5+pGADT7, and pHIS2-DrRALF6+pGADT7 did not grow on SD-TLH medium supplemented with 100 mM, 150 mM, 150 mM, and 50 mM 3-AT, respectively, indicating that the *HIS3* reporter gene was not activated under these conditions (Supplementary Figures S7 and S8).

**Figure 10:**
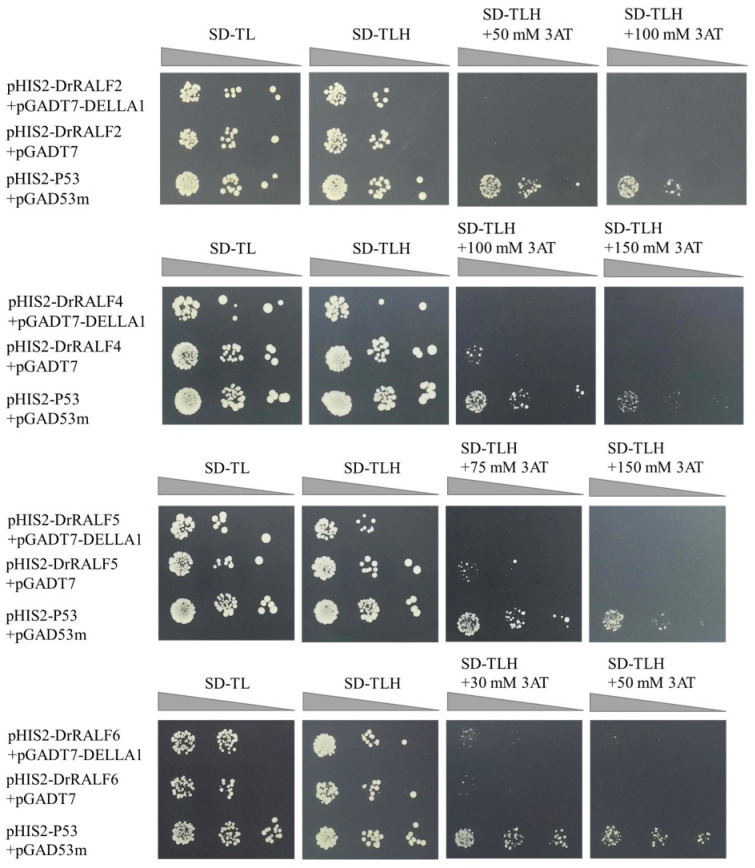
Yeast one-hybrid assay for detecting the interaction between pHIS2-DrRALF2/4/5/6 and pGADT7-DrDELLA1. The triangle represents the concentration gradient of the yeast culture on the plates, decreasing from left to right (10^0^, 10^−1^, and 10^−2^). The positive control was pHIS2-P53+pGAD53m, and the negative control was pHIS2-DrRALF2/4/5/6+pGADT7.

These recombinant plasmids were co-transformed into Y187 to assess the interaction between pHIS2-DrRALF2/4/5/6 and pGADT7-DrDELLA1 through a yeast one-hybrid assay. The positive control, pGAD53m+pHIS2-p53, grew as expected on SD-TL, SD-TLH, and SD-TLH media supplemented with 3AT (Figure 10). The negative controls, including pHIS2-DrRALF2+pGADT7, pHIS2-DrRALF4+pGADT7, pHIS2-DrRALF5+pGADT7, and pHIS2-DrRALF6+pGADT7, grew normally on SD-TL and SD-TLH media. However, they did not grow on SD-TLH media supplemented with 100 mM 3AT, 150 mM 3AT, 150 mM 3AT, and 50 mM 3AT. pHIS2-DrRALF2+pGADT7-DELLA1 exhibited normal growth on both SD-TL and SD-TLH media but did not grow on SD-TLH media supplemented with 100 mM 3AT. pHIS2-DrRALF4+pGADT7-DELLA1 and pHIS2-DrRALF5+pGADT7-DELLA1 grew normally on SD-TL and SD-TLH media but were unable to grow on SD-TLH media supplemented with 150 mM 3AT. In contrast, pHIS2-DrRALF6+pGADT7-DELLA1 grew normally on SD-TL, SD-TLH, and SD-TLH media supplemented with 50 mM 3AT. These findings demonstrate an interaction between pHIS2-DrRALF6 and pGADT7-DELLA1; pHIS2-DrRALF2, pHIS2-DrRALF4, and pHIS2-DrRALF5 did not interact with pGADT7-DELLA1.

## 3. Discussion

### 3.1. RALF Genes in Yam

RALF peptides occur widely in plants and regulate growth, hormone signaling, and stress responses. Initially identified for their inhibitory effect on primary root growth, RALFs have been shown to exert similar effects in various plants, including *Arabidopsis*, *Taraxacum koksaghyz*, and grass sugarcane [15,29,30,31]. Additional studies have revealed that RALFs regulate pollen maturation and germination, pollen tube integrity, flowering, and fruit ripening [17,22,23,32,33]. RALFs also affect plant immunity and adaptation to abiotic stress [34,35]. *RALF*s have been identified in multiple species, including *Arabidopsis*, rice, maize, and soybean [36,37,38,39]. Here, we identified seven *RALF* genes in the yam genome, which is significantly smaller than the number of *RALF* genes identified in species such as *Arabidopsis* (37), maize (20), *Chenopodium quinoa* (18), and soybean (27) [37,38,39,40]. This disparity might be related to the vegetative reproduction of yam. Vegetatively propagated plants, which lack genetic recombination, typically exhibit lower genetic diversity and have a more stable genomic structure, which potentially limits the expansion and diversification of gene families [41,42].

RALFs are typical secretory proteins, and their N-terminal contains a conserved cysteine-rich signal peptide, which is critically important for processing and quality control [43,44]. To generate a mature active peptide, the conserved RRXL motif of the RALF precursor is cleaved by a protease [45]. Previous research has indicated that the RR motif negatively regulates immune responses by RALFs [46,47]. However, not all RALF proteins possess the conserved RR motif, and only approximately one-third of the RALFs in *Arabidopsis* contain the S1P cleavage site [18,40]. In our study, all DrRALFs contained signal peptide sequences, and most of them contained the RR motif (except for DrRALF1 and DrRALF2). This suggests that DrRALFs are functionally conserved in yam.

The conserved YISY motif plays an essential role in receptor binding as well as RALF activity. The YI and Y residues are highly conserved. Among the seven DrRALFs, five contain the conserved YISY motif, suggesting that their functional activity is high. However, the YI motif is replaced by KI and TI in DrRALF2 and DrRALF5, respectively. Our findings also indicate that RALFs lacking the YISY motif were still able to bind to receptors and form complexes, but they lost their functional activity.

### 3.2. Gene Duplication and Phylogenetic Analysis Reveal Mechanisms of Gene Family Expansion and the Functional Conservation of DrRALFs

The expansion of plant gene families is closely associated with genome size, environmental adaptation, and the evolutionary history of species [48,49,50]. WGD and tandem duplication drive gene family expansion, especially in plants [51,52,53]. These duplication events not only increase the number of genes but also facilitate the functional diversification of genes. Tandem duplication has been a major driver of *RALF* gene expansion in *Arabidopsis*, soybean, and rice [38,54]. However, Xue et al. [39] indicated that WGD is the main factor underlying the expansion of *RALF* genes in maize. In our study, three of the seven *DrRALFs* identified in yam stemmed from WGD or segmental duplication, and evidence of tandem duplications is lacking. This indicates that *DrRALF* genes mainly originated from WGD or segmental duplication, in contrast to other plant species, such as *Arabidopsis* or soybean, where tandem duplication is the major driver of *RALF* gene expansion. This discrepancy may reflect the unique evolutionary history of yam. As a vegetatively propagated plant, the expansion of gene families in yam may depend more on WGD and segmental duplication, which help maintain genomic stability and enhance genetic diversity within the plant.

Campbell and Turner [55] conducted a comprehensive study on *RALF* genes across various plants and revealed that they can be classified into four groups based on their conserved motifs. Our phylogenetic analysis also categorized *RALF* genes from *Arabidopsis* and yam into four clades according to their evolutionary distances. The distribution of *DrRALF* genes was uneven, with six *DrRALF* genes clustered in Clade III. This pattern suggests that *RALF* genes in yam and other species are highly conserved, implying that the functional diversity of these *RALF* genes in yam may be relatively limited. However, the single *DrRALF* gene in Clade II may have a distinct function. Our analysis revealed that *DrRALF3* shares homology with *RALF34*, and *DrRALF2* is likely orthologous to *RALF32*, suggesting that these gene pairs may play similar roles in different species. Previous studies have demonstrated that *RALF32* and *RALF34* regulate various processes in *Arabidopsis*, including seedling growth, lateral root formation, and pollen tube rupture [17,18,56]. Future functional studies will be essential for exploring the roles of *DrRALF* genes in yam.

### 3.3. Analysis of Expression Patterns Suggests That DrRALFs Play a Role in Yam Development

RALFs are thought to be involved in both vegetative and reproductive growth processes in various plant species. In particular, they are known to inhibit root growth by limiting cell expansion [44,57]. In *Arabidopsis*, overexpression of *RALF1* induces a dwarf phenotype characterized by smaller leaves and reductions in root length and numbers of lateral roots, and silencing of *RALF1* leads to elongated roots and hypocotyls [58,59]. In our study, the root-specific expression of *DrRALF5* in yam suggests that its role in root elongation or patterning is conserved and potentially analogous to the role of RALF1 in *Arabidopsis*.

Moreover, the floral-specific expression patterns of several *DrRALF* genes (*DrRALF1–4* and *DrRALF6*) suggest they may participate in floral organ differentiation or reproductive signaling. Previous research has demonstrated that RALFs regulate flowering and fruit maturation in plants. Yuan et al. [60] found that *RALF* transcript abundance in *Phalaenopsis* leaves significantly increases during the flowering period. RALF1 delays flowering in *Arabidopsis* by interacting with the receptor FERONIA (FER), thereby regulating the accumulation of transcripts of flowering-related genes and mRNA alternative splicing [33].

RALFs have also been associated with fruit development and maturation in other species such as *Solanum chacoense* [61], Chinese cabbage [62], and strawberry [63]. The expression levels of *DrRALF1*, *DrRALF2*, *DrRALF3*, and *DrRALF6* genes were down-regulated during yam tuber development, whereas the expression of *DrRALF4* and *DrRALF5* peaked in the early stage of tuber development. These patterns imply that *DrRALFs* may play temporally and tissue-specific roles in the tuber expansion process.

Furthermore, the enrichment of hormone-responsive *cis*-regulatory elements in *DrRALF* promoters, particularly those involved in GA, ABA, and auxin signaling, suggests that these genes may act as integrators of environmental and hormonal cues during tuber development (Figure 4).

### 3.4. DrRALF6 May Be Involved in Regulating Tuber Expansion in Yam Through the GA Signaling Pathway

GA is a key hormone involved in tuber expansion that primarily affects tuber growth and development by either directly or indirectly modulating cell elongation, division, and carbohydrate metabolism [4,5,6,7,8]. Exogenous GA4/7 application promotes stolon elongation and delays tuber initiation in potatoes [9,64]. In *Helianthus tuberosus* L., GA3 levels are higher prior to tuber formation, which favors stolon formation and growth; however, during tuber development, GA3 levels gradually decrease, and they are negatively correlated with the content of dry matter and sugar in the tubers [10]. GA regulates tuber initiation in yam through the GA-GID1-DELLA signaling pathway [6,14]. We identified potential DrDELLA1-binding sites in the promoters of *DrRALF1*, *DrRALF2*, *DrRALF4*, *DrRALF5*, *DrRALF6*, and *DrRALF7*. Quantitative PCR analysis revealed that GA induced *DrRALF2*, *DrRALF4*, and *DrRALF6* expression, while PP333 suppressed it. *DrRALF5* expression was suppressed by GA but promoted by PP333. Yeast one-hybrid assays further confirmed that the *DrRALF6* promoter region interacts with DrDELLA1, suggesting that it plays a role in tuber expansion regulation via the GA-GID1-DELLA pathway. Additionally, GA can activate DELLA-independent pathways, such as calcium-dependent signaling, which suppress tuber expansion in potatoes [11,65,66]. Whether the remaining three *DrRALF* genes contribute to DELLA-independent GA pathways during tuber expansion requires additional study.

In summary, we identified and characterized seven *RALF* genes in *D. rotundata*, including their genomic distribution, evolutionary dynamics, and tissue-specific expression profiles. Notably, *DrRALF6* showed GA-responsive expression, and the protein DrDELLA1 (the GA-signaling regulator) has also been shown to bind to its promoter, suggesting a potential link between RALF signaling and gibberellin pathways. Although further in vivo functional validation is required, this interaction supports the hypothesis that DrRALF6 may play a role in GA-mediated tuber development. These findings enhance our understanding of the molecular mechanisms underlying yam tuber development and provide promising targets for molecular breeding and genetic engineering to improve yield and quality. Overall, our findings may aid the regulation of hormone-responsive pathways in yam and support molecular breeding strategies to improve tuber yield and quality.

## 4. Materials and Methods

### 4.1. Identification of RALF Genes in Yam

*RALF* genes of *Arabidopsis thaliana* were obtained from GenBank and The Arabidopsis Information Resource; further details are provided in Supplementary Table S1. *Dioscorea rotundata* data were obtained from Phytozome. A BLAST search was conducted locally using TBtools (v1.108) to identify potential *DrRALF* genes; queries were conducted using *AtRALF* sequences [67]. The E-value threshold was set to 1e-5; the maximum number of sequences retrieved during the initial search (Num of Hits) was 500, and the number of sequences considered for final alignment in the subsequent steps was 250. The initial round of analysis identified seven candidate sequences, which were further subjected to BLAST searches against the SwissProt protein database (https://www.uniprot.org/uniprotkb?query=reviewed:true, accessed on 31 January 2023). These sequences were classified as *DrRALF* genes (Table 1).

### 4.2. Chromosomal Location and Collinearity Analysis of DrRALF Genes

The genomic locations of genes were obtained from the Phytozome database. The data were filtered using TBtools (v1.108), and their chromosomal distribution was visualized using TBtools (Advanced Circos module of TBtools v1.108). Collinearity analysis of *DrRALFs* was conducted using MCScanX in TBtools (v1.108) with default settings.

### 4.3. Properties of DrRALF Proteins

The molecular weight (Da) and isoelectric point (pI) were determined by the Protein Parameter Calc of TBtools (v1.108)) based on the full-length proteins.

### 4.4. Phylogenetic Analysis of DrRALF Proteins in Yam

*DrRALF* protein sequences from both yam and *Arabidopsis* were aligned by ClustalW in MEGA 11.0.13 with default parameters. The optimal substitution model was selected in MEGA 11.0.13, and the tree was generated using the maximum-likelihood method with the JTT+G model and 1000 bootstrap iterations. iTOL v6 (https://itol.embl.de/, accessed on 18 March 2023) was used to visualize the tree.

### 4.5. Promoter Cis-Regulatory Element Analysis

*Cis*-regulatory elements of the *DrRALF* promoters were identified using the PlantCARE database (https://bioinformatics.psb.ugent.be/webtools/plantcare/html/, accessed on 7 February 2023). *Cis*-elements potentially associated with key biological processes were retained. Further details are provided in Supplementary Table S2. TBtools (Simple Biosequence Viewer of TBtools v1.108) was used to visualize the *cis*-elements, which ensured that the regulatory sequences were comprehensively analyzed.

### 4.6. Gene Expression Analysis

Transcriptome datasets for yam *DrRALFs* for different tissues and treatments were obtained from the Sequence Read Archive (SRA) database under accession numbers SRP152752 and DRP003729. The SRP152752 dataset comprises yam tuber tissue samples collected at different developmental stages (initiation, early, middle, and mature), with three biological replicates per stage [46]. DRP003729 consists of transcriptomes derived from various yam tissues [68]. These transcriptome datasets were downloaded and converted into FASTQ format. Transcript abundance was quantified using the Kallisto Super GUI Wrapper (TBtools v1.108) and a pseudo-alignment algorithm. The raw reads were pseudo-aligned to the yam reference transcriptome, and their expression values were normalized to transcripts per million (TPM). Next, the normalized data were extracted, log_2_-transformed, and visualized via a heatmap generated with Table Row Extract or Filter (TBtools v1.108).

### 4.7. Quantitative Real-Time PCR (qRT-PCR) Validation

The yam cultivar GH16 (Guihuai 16) was cultivated during 2022–2023, and its germination and growth characteristics were as previously outlined by Zhou et al. [6]. To characterize the expression variation in *DrRALFs* at different stages of tuber development, tubers were collected at five developmental stages: the initial (40 d), early expansion (60 d), middle expansion (120 d), late expansion (150 d), and maturation (180 d) stages. Five plants were randomly selected for each replication.

To evaluate the effect of gibberellin and an inhibitor of its biosynthesis (PP333) on the expression of *DrRALF* genes, foliar treatments were performed by spraying GA_3_ and PP333 at a concentration of 200 mg/L, as described by Zhou et al. [6]. Water was used as the control (CK). Tubers were collected 5 d after treatment. The distal ends (5 mm) of five tubers were washed, chopped, and pooled to form one biological replicate. After freezing in liquid nitrogen, all samples were maintained at −80 °C.

Based on the rna-XM_039267626.1 (*DrRALF1*), rna-XM_039269945.1 (*DrRALF2*), rna-XM_039268398.1 (*DrRALF3*), rna-XM_039271549.1 (*DrRALF4*), rna-XM_039273644.1 (*DrRALF5*), and rna-XM_039258130.1 (*DrRALF6*) sequences in the yam genome, the gene-specific primers for *DrRALFs* were designed using Primer Premier 5. Primer specificity was verified using NCBI BLAST, with *DrACTIN* used as the reference gene. Primer sequences are shown in Supplementary Table S3. ArtiCanATM SYBR qPCR Mix from Tsingke Biotechnology Co., Ltd. (Beijing, China) was used to perform qRT-PCR. The relative expression levels of *DrCrRLK1Ls*, with three independent biological replicates, were calculated using the 2^−ΔΔCT^ method.

### 4.8. Verification of the Secretory Function of DrRALFs

The yeast strain YTK12 from Nanjing Ruiyuan Biotechnology Co., Ltd. (Nanjing, China) was used to confirm the secretory properties of DrRALF2, DrRALF4, DrRALF5, and DrRALF6. The coding sequences of these *DrRALF* genes were cloned into the pSUC2 vector. Transformation of the pSUC2-DrRALF constructs into YTK12 was performed following Qiao et al. [69]. Yeast growth was assessed on CMD-W and YPRAA media, and color reactions were monitored using 2, 3, 5-triphenyltetrazolium chloride (TTC).

### 4.9. Yeast One-Hybrid Assay

Yeast one-hybrid assays were performed to validate the interactions between DrDELLA1 and the promoter regions of *DrRALFs*. The 2000 bp upstream promoter regions of *DrRALF2*, *DrRALF4*, *DrRALF5*, and *DrRALF6* were inserted into the *Eco*RI-*Sac*I, *Eco*RI-*Xho*I, *Eco*RI-*Sac*I, and *Eco*RI-*Sac*I sites of the pHIS2 vector, respectively. DrDELLA1 was fused into the *Eco*RI-*Sac*I site of the pGADT7 prey vector. The assay followed the protocols outlined in the *PT3024-1/Yeast Protocols Handbook* (Clontech, US). Both the bait and prey vectors were co-transformed into *Saccharomyces cerevisiae* strain Y187. After overnight cultivation in YPDA medium, the cells were transferred to fresh medium and co-transformed with the plasmids. After transformation, yeast cells were plated on selective SD-TL medium (without tryptophan and leucine) to select for successful co-transformants. Positive interaction candidates were further selected by plating increasing concentrations of 3-aminotriazole (3-AT), a competitive inhibitor of HIS3 expression, onto SD-TLH medium (lacking tryptophan, leucine, and histidine). The *HIS3* reporter gene, which was activated following successful protein–protein interactions, was used to detect interactions between DrDELLA1 and the *DrRALF* promoter regions. The assay was repeated in triplicate to ensure reproducibility, with both positive and negative controls included to validate the specificity and reliability of the interactions.

### 4.10. Statistical Analysis

All experiments were performed with at least three biological replicates to ensure statistical reliability. Quantitative real-time PCR (qRT-PCR) data were processed using the 2^−ΔΔCT^ method, and differences in expression levels between treatments or developmental stages were analyzed using Student’s *t*-test in Microsoft Excel (2019).

## Figures and Tables

**Table 1:** Information on the seven identified RALFs in yam.

GeneID	Chr ID	Gene Range	Gene Length (bp)	Num of mRNA	mRNA ID	Num of Exon	mRNA Range	mRNA Length (bp)	Rename
gene-LOC120260185	NC_052475.1	26172276:26172596	321	1	rna-XM_039267626.1	1	26172276:26172596	321	DrRALF1
gene-LOC120261923	NC_052475.1	26020100:26020822	723	1	rna-XM_039269945.1	1	26020100:26020822	723	DrRALF2
gene-LOC120260830	NC_052475.1	1661275:1661934	660	1	rna-XM_039268398.1	1	1661275:1661934	660	DrRALF3
gene-LOC120263587	NC_052476.1	20662702:20663395	694	1	rna-XM_039271549.1	1	20662702:20663395	694	DrRALF4
gene-LOC120265695	NC_052477.1	2075500:2076092	593	1	rna-XM_039273644.1	1	2075500:2076092	593	DrRALF5
gene-LOC120249577	NC_052489.1	30005811:30006480	670	1	rna-XM_039258130.1	1	30005811:30006480	670	DrRALF6
gene-LOC120254762	NW_024087005.1	33774:34492	719	1	rna-XM_039262807.1	1	33774:34492	719	DrRALF7

**Table 2:** Protein parameters of DrRALFs.

Sequence ID	Number of Amino Acids	Molecular Weight	Theoretical pI	Instability Index	Aliphatic Index	Grand Average of Hydropathicity
DrRALF1	106	11,639.64	6.55	48.34	68.02	0.029
DrRALF2	124	13,686.3	8.18	49.1	59.11	−0.596
DrRALF3	126	14,165.94	8.4	47.68	67.38	−0.316
DrRALF4	111	12,165.91	8.63	49.55	83.69	−0.078
DrRALF5	127	14,218.17	9.02	51.96	90.79	−0.398
DrRALF6	117	12,613.46	9.58	48.61	82.74	−0.056
DrRALF7	128	14,308.57	9.83	47.7	73.98	−0.359

## Data Availability

All primary data supporting the findings of this study are openly available in the NCBI SRA under accession numbers SRP152752, (https://www.ncbi.nlm.nih.gov/sra/?term=SRP152752, accessed on 10 February 2023) and DRP003729 (https://www.ncbi.nlm.nih.gov/sra/?term=DRP003729, accessed on 10 February 2023). All data generated or analyzed in this study are included in this published article (Supplementary Data).

## Supplementary Materials

The following supporting information can be downloaded at https://www.mdpi.com/article/10.3390/ijms26136151/s1.

## Abbreviations

The following abbreviations are used in this manuscript:

GA	Gibberellin
qRT-PCR	Quantitative Real-Time PCR
RALF	Rapid Alkalinization Factor
WGD	Whole-Genome Duplication
PP333	Paclobutrazol
