# Supplementary figures and images for: Genome-Wide Characterization of DrRALF Genes in Yam (Dioscorea rotundata) Reveals Their Potential Roles in Tuber Expansion and the Gibberellin Response
Source: Int J Mol Sci. 2025 Jun 26;26(13):6151. doi: 10.3390/ijms26136151 (PMC12249873; doi:10.3390/ijms26136151)

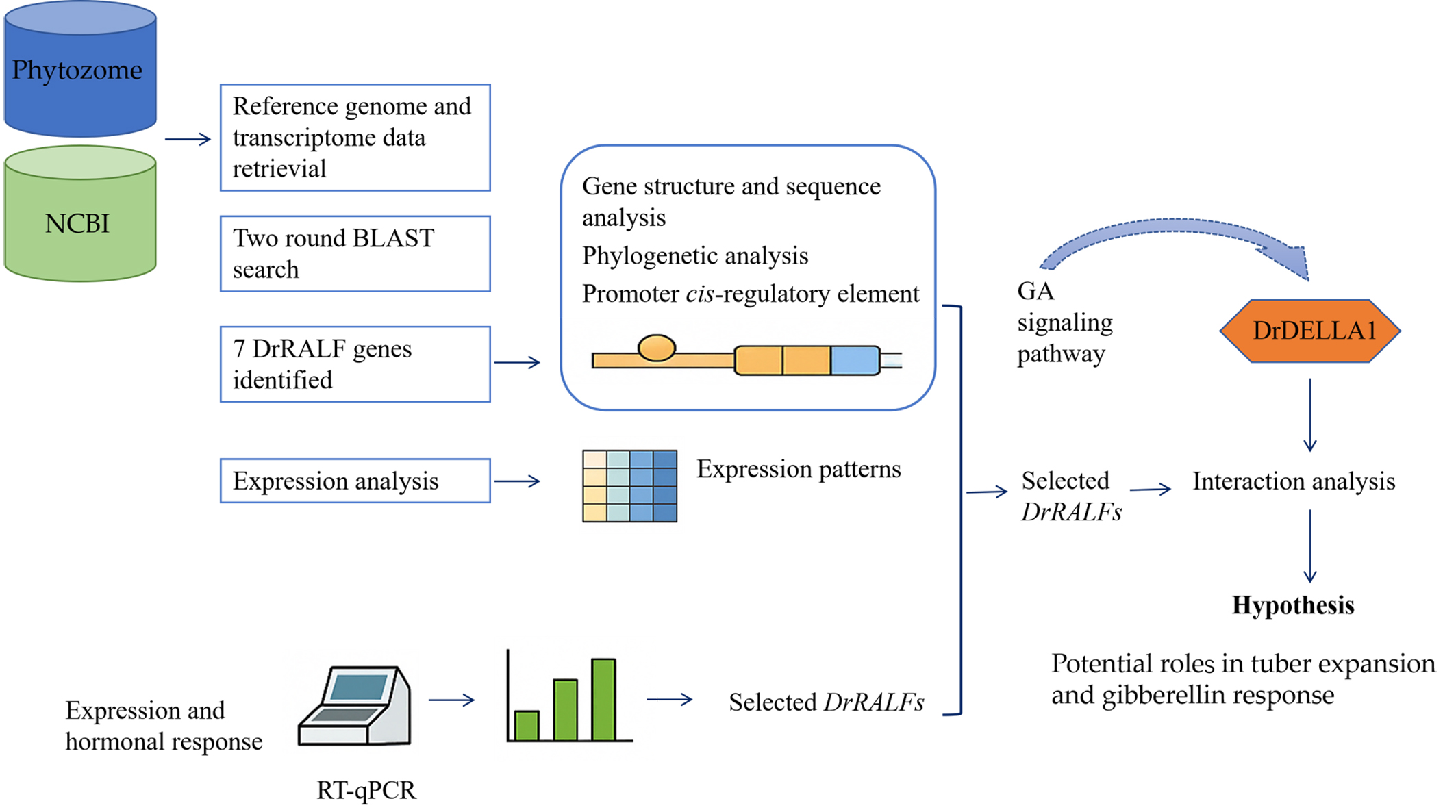

Supplement: Supplementary file 1 [file ijms-26-06151-s001.zip › Supplementary Fig S1.jpg]

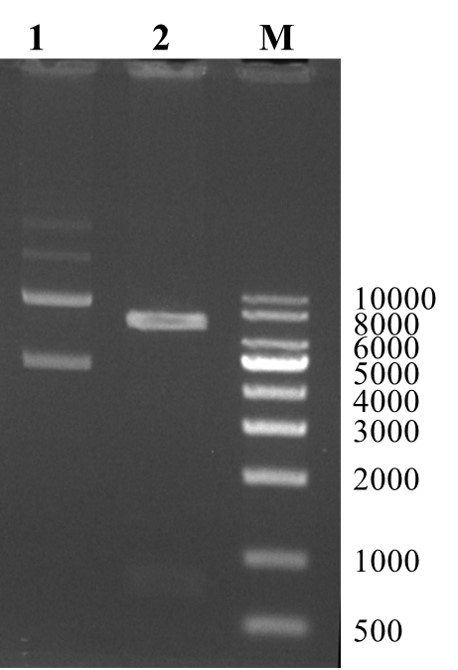

Supplement: Supplementary file 1 [file ijms-26-06151-s001.zip › Supplementary Fig S2.jpg]

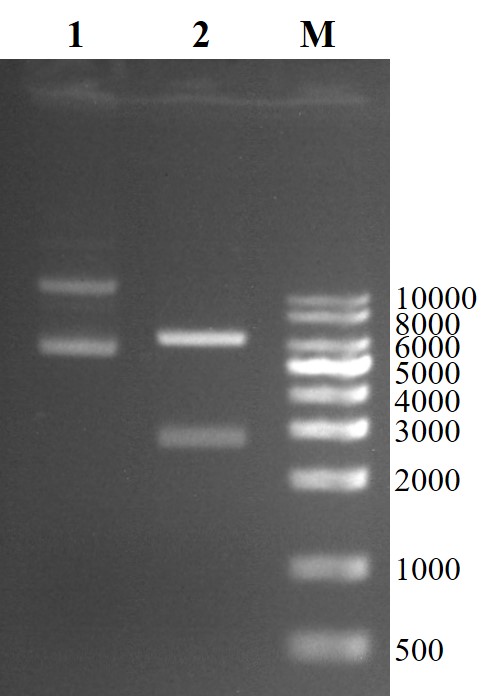

Supplement: Supplementary file 1 [file ijms-26-06151-s001.zip › Supplementary Fig S3.jpg]

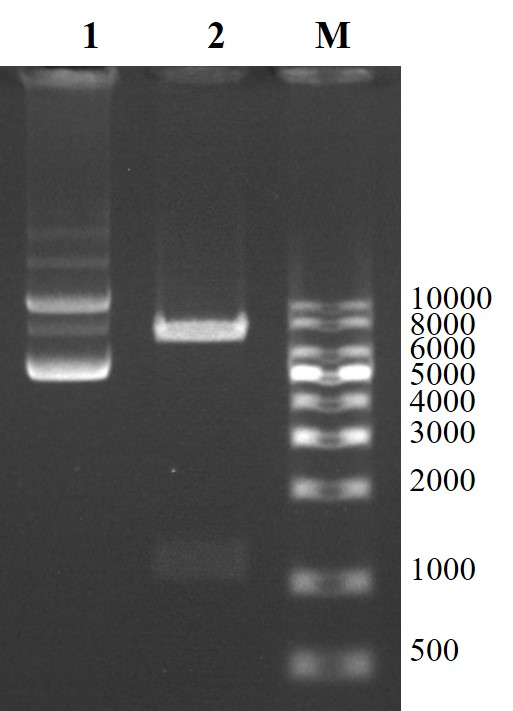

Supplement: Supplementary file 1 [file ijms-26-06151-s001.zip › Supplementary Fig S4.jpg]

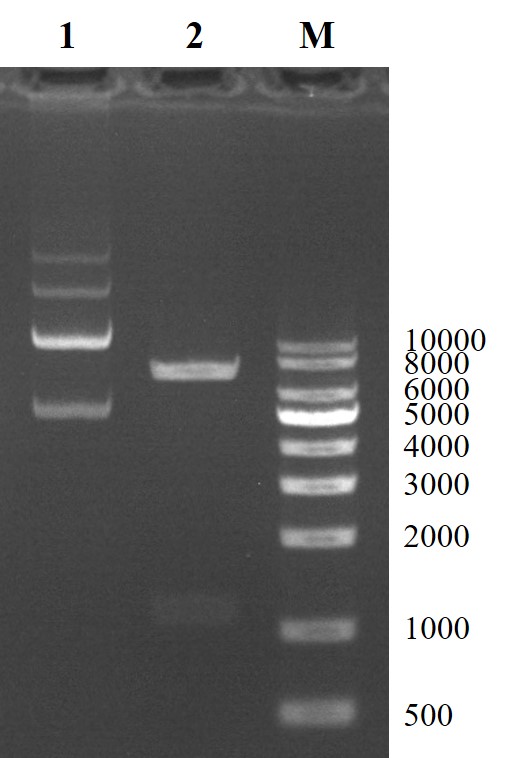

Supplement: Supplementary file 1 [file ijms-26-06151-s001.zip › Supplementary Fig S5.jpg]

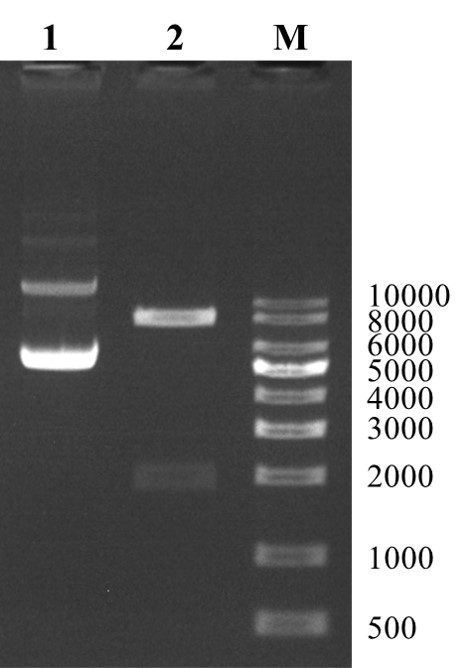

Supplement: Supplementary file 1 [file ijms-26-06151-s001.zip › Supplementary Fig S6.jpg]

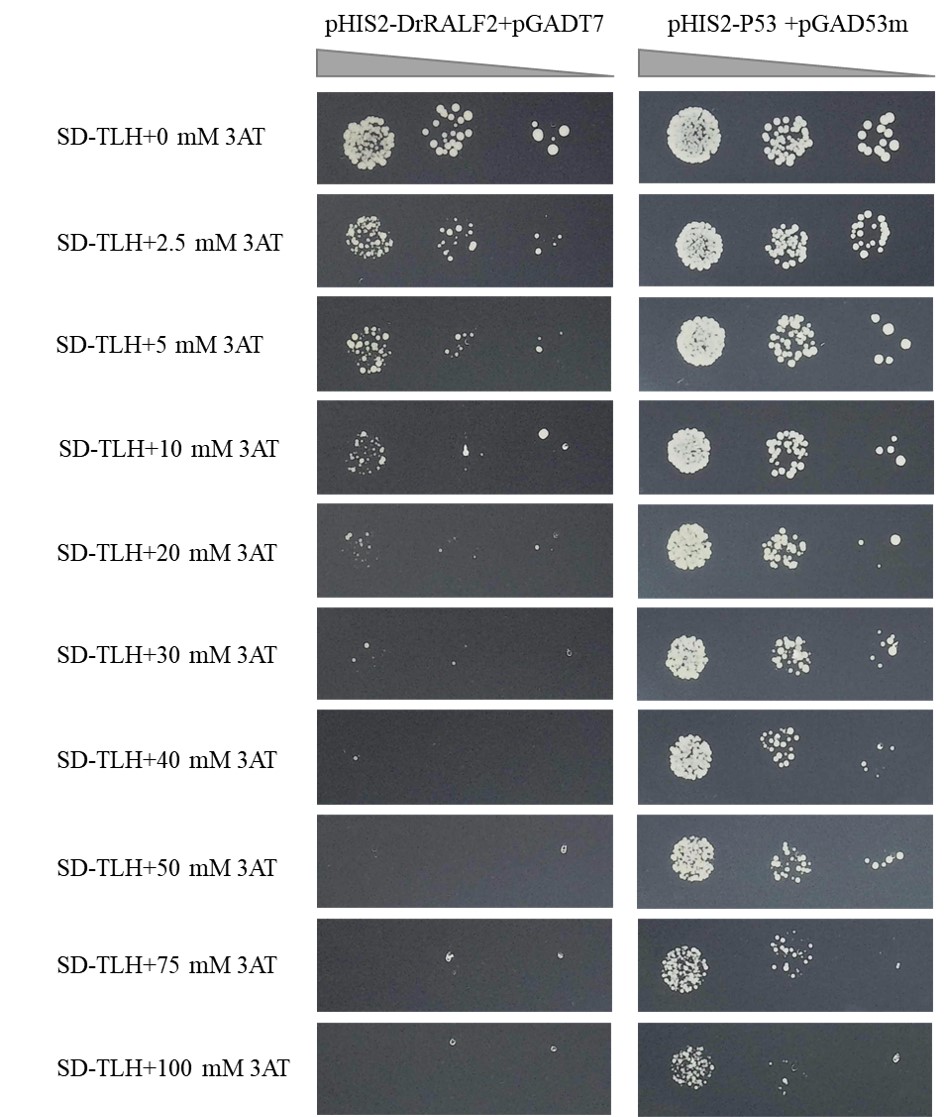

Supplement: Supplementary file 1 [file ijms-26-06151-s001.zip › Supplementary Fig S7.jpg]

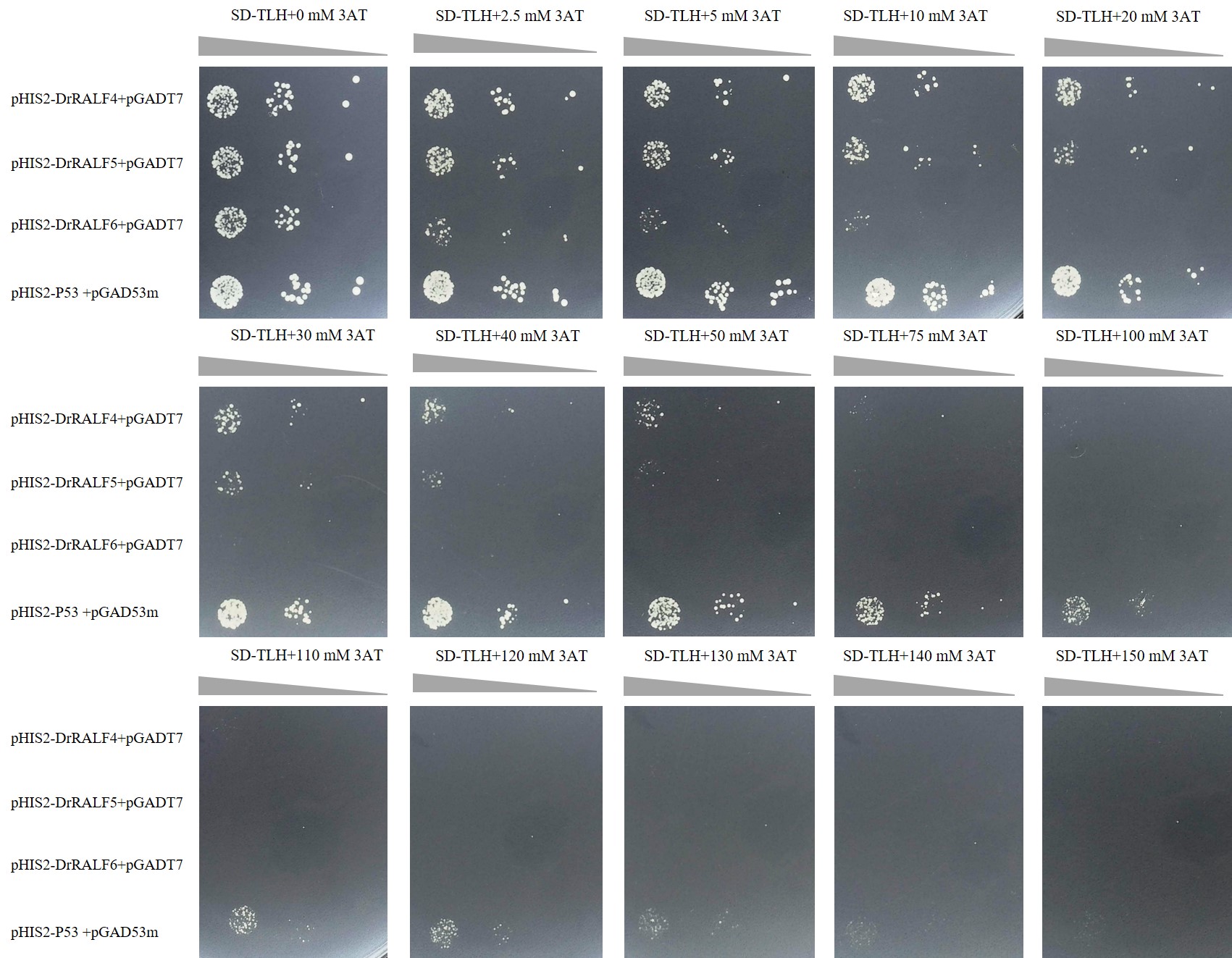

Supplement: Supplementary file 1 [file ijms-26-06151-s001.zip › Supplementary Fig S8.jpg]
